# Milan criteria in the MELD era—is it justifiable to extend the limits for orthotopic liver transplantation?

**DOI:** 10.1186/s12957-020-01932-6

**Published:** 2020-07-07

**Authors:** Mehmet Haluk Morgul, Philipp Felgendreff, Andreas Kienlein, Ulrich Gauger, Katrin Semmling, Hans-Michael Hau, Hans-Michael Tautenhahn, Michael Bartels

**Affiliations:** 1 Department of Visceral, Transplant, Thoracic, and Vascular Surgery, University of Leipzig, Leipzig, Germany; 2 Department of General, Visceral and Transplantation Surgery, University of Münster, Münster, Germany; 3 Department of General, Visceral, and Vascular Surgery, University of Jena, Am Klinikum 1, 07749 Jena, Germany; 4 Research Programme “Else Kröner-Forschungskolleg AntiAge”, University of Jena, Jena, Germany; 5 Department of Urology and Pediatric Urology, Schwarzwald-Baar-Klinikum, Villingen-Schwenningen, Germany; 6 Private Statistical Office, Berlin, Germany; 7 Department of Gastr., Thoracic and Vascular Surgery, University Hospital Carl Gustav Carus, Technische Universität Dresden, Dresden, Germany; 8 Department for General Visceral, Thoracic and Vascular Surgery, Helios Park-Klinikum Leipzig, Leipzig, Germany

**Keywords:** HCC, Liver transplantation, Milan criteria, Up-to-seven criteria, Asan criteria, AFP score

## Abstract

**Background:**

The Milan criteria (MC) are widely used for the indication of liver transplantation (LTx) in hepatocellular carcinoma (HCC). Good long-term results have also been reported following LTx for patients exceeding the MC. In this article, we compare the overall and recurrence-free survival of our patients fulfilling and exceeding the MC according to the post-transplant histopathological results.

**Patients and methods:**

Data from 120 patients with HCC (22 females and 98 males) were analyzed. The median patient age was 61 years (Q1, Q3 54.7, 65.4), and the median MELD score was 11 (Q1, Q3 8, 15). The median follow-up period was 53 months (Q1, Q3 16.6, 78). Patients were categorized into established criteria (MC, up-to-seven (UTS), Asan criteria, AFP score), and the outcome of the individual groups was compared.

**Results:**

Seventy-four of 120 patients fulfilled the MC, 86 patients met the UTS criteria, 85 patients fulfilled the Asan criteria, and 79 patients had an AFP score less than or equal to 2. The 1- and 5-year survival rates of all patients were 76.7% and 55.6%, respectively. In total, 14.2% of all patients (5.4% of patients who met the MC, 7% of patients who met the UTS criteria, 5.9% of patients who met the Asan criteria, and 6.3% of patients who had an AFP score less than 2) experienced recurrence.

**Conclusions:**

The outcomes of the patients were comparable to those reported in the current literature. In our population, similar recurrence and survival rates of the patients were noted for patients fulfilling the UTS criteria irrespective of fulfilling or exceeding the MC. Consequently, we consider using UTS criteria as the extended criterion for LTx indication.

## Introduction

Liver transplantation (LTx) offers the only curative option for patients suffering from end-stage liver disease. In cases of hepatocellular carcinoma (HCC) with underlying liver cirrhosis, the indication for LTx is a major challenge due to the complicated natural course of this disease. To achieve an adequate distribution of the limited number of donor organs for the patients on the waiting list, it is important to identify the patients who benefit most from LTx with a sufficient prognosis.

Based on tumor-specific characteristics, numerous systems have been established for preoperative assessment of the prognosis of patients with HCC and to verify the indication for LTx [1, 2]. In this regard, the most common selection systems are based on the radiological characterization of HCC tumors. Worldwide, the Milan criteria (MC) are a well-known and widely accepted algorithm for the indication of LTx in patients with HCC. These criteria are based on the work of Mazzaferro et al., who examined the data of 48 patients with HCC and liver cirrhosis over a median follow-up period of 26 months (range 9–54 months) [3]. These very strict criteria were extended by the same group. The up-to-seven (UTS) criteria (sum of the largest tumor diameter in centimeters and the number of tumors [4]) showed similar outcomes following LTx while liberating the criteria for the indication for LTx.

Although tumor biology seems to be a better predictor of recurrence and survival following transplantation, in the current practice, tumor biology is barely involved in decision-making since there is no clinically proven biomarker for this purpose. Only higher alpha-fetoprotein (AFP) levels lead clinicians to be restrictive regarding transplantation [5]. In a recent study, Duvoux et al. proposed a mathematical algorithm based on the AFP level, tumor size, and number to assess patient survival and the probability of recurrence following LTx [6]. In a cohort of 435 patients, they showed 5-year recurrence rates of 8.8% vs 50.6% for patients with AFP scores ≤ 2 and > 2, respectively. Simultaneously, the 5-year survival rates were 67.8% and 47.5%, respectively.

Many studies have evaluated the success of LTx beyond the MC-based radiological and biological findings of patients [6–8]; however, a system extending the MC has not been solely adopted in the transplant society.

Here, we introduce the factors influencing the outcome of LTx in patients with HCC fulfilling and exceeding the MC according to the posttransplant histopathological results. Furthermore, we retrospectively analyzed our cohort for extension of the MC using the UTS, as our institutional standard policy, and the AFP score as a novel system involving tumor biology and the Asan criteria, a post-transplant histological-based prognosis system introduced by Lee et al.

## Methods

### Study design

All patients who underwent orthotopic liver transplantation between 1994 and 2013 were enrolled in the database of the Department of Visceral, Transplant, Thoracic and Vascular Surgery at the University of Leipzig. Data were collected and analyzed following approval by the university ethics committee (application number: 078-14-10032014).

Patients without histological evidence of HCC in posttransplant pathology or patients receiving a living donor transplant, secondary liver transplant, or pediatric transplant were excluded from the study. To analyze the overall survival and recurrence-free survival, all patients were classified according to four different scoring systems (MC [3], UTS [4], AFP score [6], Asan [8]) based on the postoperative histological result.

### Examination and analysis of laboratory parameters

The biochemical parameters of the patients were obtained from clinical charts, and the model for end-stage liver disease (MELD) score for each patient was calculated with the most recent parameters prior to transplantation for each patient and was calculated if the parameters necessary for determining the MELD score (bilirubin, creatinine, international normalized ratio of the same sample, receiving renal replacement therapy more than twice in the last week) were available. The most recent AFP level of the patient before transplantation was considered for the analysis.

#### HCC diagnosis

The diagnosis of HCC is confirmed only on histological examination. For the assessment of the MC [3], UTS criteria [4], Asan criteria [8], and AFP score [6], the histological results of the explanted livers showing the tumor diameter and number were used. The AFP score was calculated using the simplified algorithm described by Duvoux et al. [6].

### Postoperative immunosuppression

The standard immunosuppression regimen was based on a triple immunosuppression regimen based on calcineurin inhibitors, mycophenolic acid, and steroids immediately after transplantation. None of the patients received induction therapy. Due to the side effects of calcineurin inhibitors on some patients, a mammalian target of rapamycin inhibitor was added to the therapy no earlier than 4 weeks after transplantation.

### HCC-specific follow-up

For specific HCC follow-up after transplantation, AFP levels were measured at least twice yearly combined with an abdominal ultrasound. MRI and CT scans were routinely performed once a year in the first 5 years after transplantation and whenever suspicious AFP levels or ultrasound findings were noted.

#### Statistical analyses

Data are presented as the mean and standard deviation (normally distributed variables) or as the median and lower and upper quartile (non-normally distributed variables). For categorical variables, absolute and relative frequencies are given. The analyses were performed using R ver. 3.6 (R-Core Team). The Shapiro-Wilk test was used to prove a Gaussian distribution. To compare continuous variables, either the *t* test or the Wilcoxon rank-sum test was chosen. For categorical variables, Fisher’s exact test was carried out. Patient survival is demonstrated on Kaplan-Meier curves with the log-rank test. Cox regression and logistic regression were used for multivariate analyses. A *p* value less than 0.05 was considered significant.

## Results

### Patient demographics

During the study period, 816 liver transplantations were performed. A total of 120 patients (22 females and 98 males) showing clear evidence of HCC in posttransplant pathology were included in the analysis, with a median age of 61 years (Q1, Q3 54.7, 65.4). Twenty-six patients underwent transplantation before the MELD scoring system was implemented in Germany. The calculated median MELD score was 11 (Q1, Q3 8, 15). The most frequent causes of liver cirrhosis were alcoholic liver disease (ALD) and viral hepatitis (*n* = 75 and 21, respectively). The median waiting time was 222 days (Q1, Q3 72.5, 347 days) (Table 1).

**Table 1:** Patient demographics

	[All], *N* = 120	Number
Age	61 [54.7; 65.4]	120
< 65	91 (75.8%)	
> 65	29 (24.2%)	
Sex		120
F	22 (18.3%)	
M	98 (81.7%)	
Disease		
ALD	75 (62.5%)	
HCV	14 (11.7%)	
HBV	7 (5.8%)	
Cryptogen	12 (10%)	
Other	12 (10%)	
AFP (ng/ml)	8.00 [4.10; 70.0]	105
Waiting_time (days)	212 [72.5; 347]	120
*D* (mm)	33.2 (20.8)	120
MELD	11 [8; 15]	97
MELD ≤ 15	71 (73.2%)	
MELD = 15–30	17 (17.5%)	
MELD > 30	9 (9.28%)	
preTreat		120
No pre-treatment	45 (37.5%)	
Pre-treatment	75 (62.5%)	
*N*		100
*N* = 1	59 (59.0%)	
*N* = 1–3	30 (30.0%)	
*N* > 3	11 (11.0%)	
Grade		59
1	23 (39.0%)	
2	33 (55.9%)	
3	3 (5.08%)	
Asan		120
Exceeding	35 (29.2%)	
Fulfilling	85 (70.8%)	
AFP score		105*
> 2	26 (24.8%)	
≤ 2	79 (75.2%)	
MC		120
Exceeding	46 (38.3%)	
Fulfilling	74 (61.7%)	
UTS		120
Exceeding	34 (28.3%)	
Fulfilling	86 (71.7%)	

*ALD* alcoholic liver disease, *HCV* hepatitis C virus, *HBV* hepatitis B virus, *AFP* alpha-feto protein in ng/ml, *D* diameter of the largest tumor in mm, MELD model of end-stage liver disease, *N* number of tumors, *MC* Milan criteria, *UTS* up-to-seven criteria

*Missing AFP values for 15 patients. Data are shown as the average and standard deviation (round brackets) for normally distributed data and as the median and quantiles (square brackets) for non-normally distributed data

### HCC diagnosis

Eighty-nine patients received a diagnosis of HCC prior to transplantation based on a biopsy or MRI and CT scan, and the diagnosis was proven by postoperative pathology. Incidental HCC was detected in 31 patients according to the postoperative pathological examination. In our population, biopsy was performed preoperatively in 46 cases, proving HCC in 36 patients with 78.2% accuracy, followed by MRI with 68.6% accuracy (35 out of 51 patients) and CT with 64.3% accuracy (72 out of 112 patients). In total, 62.5% of all patients received transarterial chemoembolization as bridging therapy, followed by radiofrequency ablation or both. According to the postoperative histological results, 74 of 120 patients met the MC, 86 patients met the UTS criteria, and 85 patients met the Asan criteria. Out of 46 MC-exceeding patients, 12 patients fulfilled UTS and 11 patients fulfilled the Asan criteria.

The AFP score could be calculated only for 105 patients due to the missing AFP values of 15 patients. A total of 79 patients had an AFP score equal to or less than 2. Only five patients exceeding the MC had an AFP score equal to or less than 2.

### HCC recurrence-free survival

Seventeen of the 120 patients (14.2%) developed HCC recurrence after a median of 28 months following transplantation (range 5–63 months). According to the univariate analyses, the parameters sex, age (≤ or > 65 years), underlying disease, MELD score, and pretreatment were not associated with HCC recurrence in our cohort. Only the AFP level (*p* = 0.009), number of tumors (*p* = 0.012), maximum diameter of the largest tumor (*p* = 0.015), and tumor grade (*p* = 0.045) were related to HCC recurrence.

**Fig. 1:**
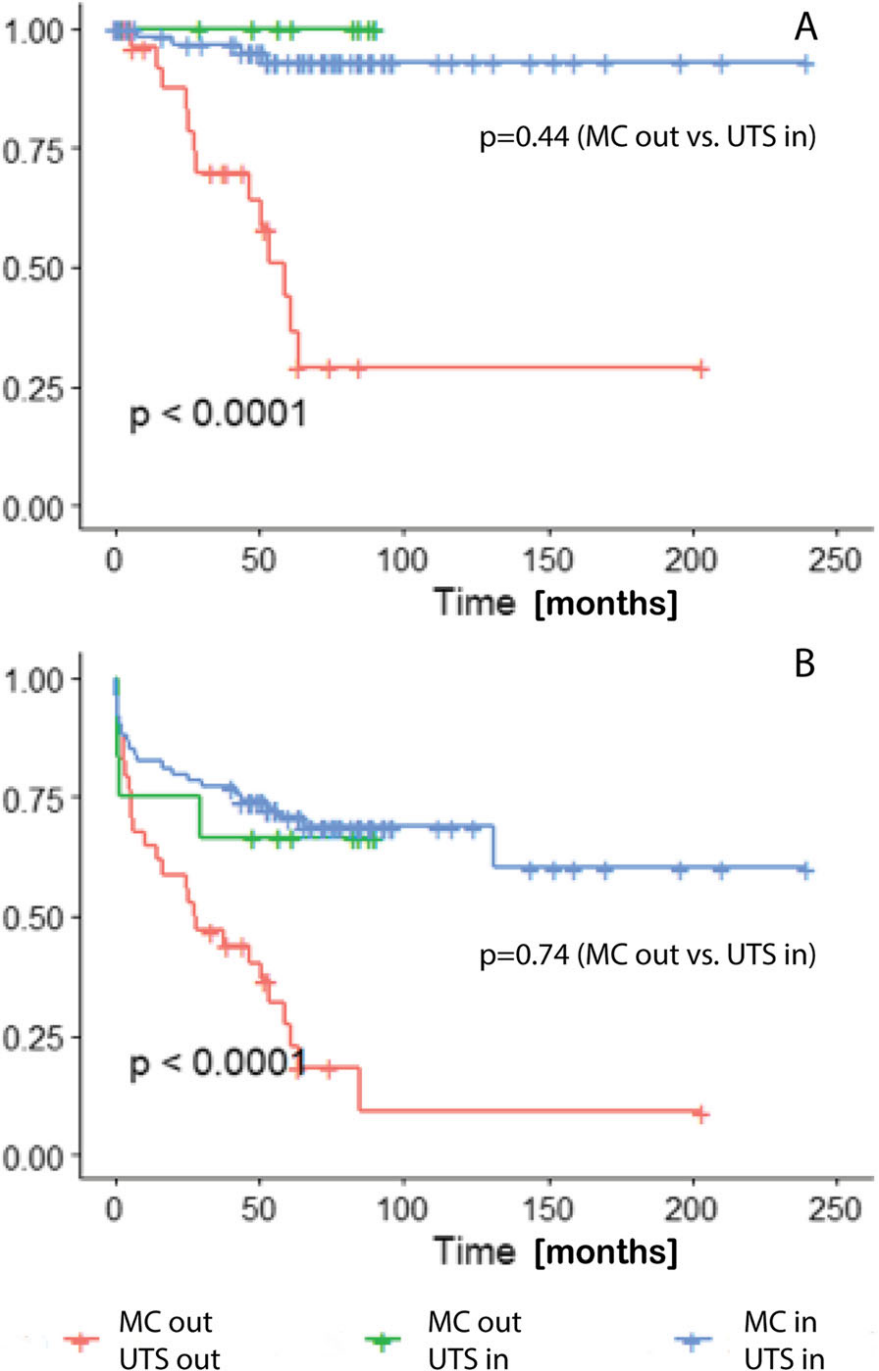
Kaplan-Meier curves for the HCC recurrence (**a**) and patient survival (**b**). Red line for patients exceeding MC and UTS, blue line for patients fulfilling MC and UTS, and green line for patients exceeding MC but fulfilling UTS

Four of 74 (5.4%) patients fulfilled the MC criteria, four of 86 (4.7%) patients met the UTS criteria, and five of 85 (5.9%) patients fulfilled the Asan criteria and developed HCC recurrence. Five of 79 (6.3%) patients with an AFP score of 2 or less experienced HCC recurrence. These criteria were highly significant in the distinction between patients according to disease recurrence (MC *p* = 0.001, UTS criteria *p* < 0.001, Asan criteria *p* < 0.001, AFP score *p* = 0.009). For the prediction of HCC recurrence, the sensitivity and specificity for the MC were 76% and 68%, respectively; for the UTS criteria, 76% and 80%, respectively; for the Asan criteria, 71% and 78%, respectively; and for the AFP score, 71% and 84%, respectively (Table 2). However, in the multivariate analyses, none of the parameters nor the scoring systems reached statistical significance (data not shown). According to the Kaplan-Meier curves, patients fulfilling UTS but exceeding the MC showed no significant changes in HCC recurrence (Fig. 1).

**Table 2:** Univariate association analysis for HCC recurrence

	HCC, *N* = 17	No HCC, *N* = 103	*p* overall
Age 65			0.357
<65	11 (64.7%)	80 (77.7%)	
> 65	6 (35.3%)	23 (22.3%)	
Sex			0.735
F	2 (11.8%)	20 (19.4%)	
M	15 (88.2%)	83 (80.6%)	
AFP (ng/ml)	165 [6.70; 435]	7.10 [4.00; 39.1]	0.009
Waiting_time (days)	230 [31.0; 394]	211 [75.5; 344]	0.934
*D* (mm)	43.4 (20.0)	31.5 (20.6)	0.035
MELD			1.000
MELD ≤ 15	12 (80.0%)	59 (72.0%)	
MELD = 15–30	2 (13.3%)	15 (18.3%)	
MELD > 30	1 (6.67%)	8 (9.76%)	
preTreat			1.000
No pre-treatment	6 (35.3%)	39 (37.9%)	
Pre-treatment	11 (64.7%)	64 (62.1%)	
*N*			0.012
*N* = 1	1 (12.5%)	58 (63.0%)	
*N* = 1–3	5 (62.5%)	25 (27.2%)	
*N* > 3	2 (25.0%)	9 (9.78%)	
Grade			0.045
1	0 (0.00%)	23 (43.4%)	
2	5 (83.3%)	28 (52.8%)	
3	1 (16.7%)	2 (3.77%)	
Asan			< 0.001
Exceeding	12 (70.6%)	23 (22.3%)	
Fulfilling	5 (29.4%)	80 (77.7%)	
AFP score			< 0.001
> 2	12 (70.6%)	14 (15.9%)	
≤ 2	5 (29.4%)	74 (84.1%)	
MC			0.001
Exceeding	13 (76.5%)	33 (32.0%)	
Fulfilling	4 (23.5%)	70 (68.0%)	
UTS			< 0.001
Exceeding	13 (76.5%)	21 (20.4%)	
Fulfilling	4 (23.5%)	82 (79.6%)	

Data are shown as the average and standard deviation (round brackets) for normally distributed data and as the median and quantiles (square brackets) for non-normally distributed data

*AFP* alpha-feto protein in ng/ml, *D* diameter of the largest tumor in mm, *MELD* model of end-stage liver disease, *N* number of tumors, *MC* Milan criteria, *UTS* up-to-seven criteria

### Overall survival

The overall survival rates were 76.7% for 1 year, 67.2% for 3 years, and 55.6% for 5 years. In total, 53 patients died during the follow-up period, most of them (*n* = 17) due to HCC recurrence. Fifteen patients died from sepsis and multi-organ failure. The third most common cause of death was cardiopulmonary complications. The univariate analyses of sex, age (≤ or > 65 years), primary disease, MELD score, pretreatment, tumor diameter, number of tumors, and grade showed no statistically significant difference between survivors and non-survivors. Only AFP levels (*p* < 0.001) showed significant differences between the groups.

The 1-, 3-, and 5-year survival rates for patients fulfilling the MC were 82%, 77%, and 69%, respectively; those for patients fulfilling the UTS criteria were 81%, 76%, and 71%, respectively; those for patients fulfilling the Asan criteria were 81%, 74%, and 70%, respectively; and those for patients with an AFP score ≤ 2 were 82%, 75%, and 52%, respectively. Similar to HCC recurrence, the criteria were also highly significant regarding survival (MC: *p* = 0.001, UTS criteria: *p* < 0.001, Asan criteria: *p* < 0.001, AFP score: *p* = 0.002). The sensitivity and specificity were 57% and 76%, respectively, for the MC; 49% and 88%, respectively, for the UTS criteria; 47% and 85%, respectively, for the Asan criteria; and 41% and 87%, respectively, for the AFP score (Table 3). Again, according to the Kaplan-Meier curves, patients fulfilling UTS but exceeding the MC showed no significant changes in survival (Fig. 1). However, in the multivariate analyses, none of the parameters nor the scoring systems reached statistical significance (data not shown).

**Table 3:** Univariate association analysis of survival

	Non-survivor, *N* = 53	Survivor, *N* = 67	*p* overall
Age 65			0.248
< 65	37 (69.8%)	54 (80.6%)	
> 65	16 (30.2%)	13 (19.4%)	
Sex			1.000
F	10 (18.9%)	12 (17.9%)	
M	43 (81.1%)	55 (82.1%)	
AFP (ng/ml)	36.1 [5.40; 234]	6.20 [3.50; 26.5]	< 0.001
Waiting_time (days)	236 [77.0; 370]	198 [64.5; 316]	0.319
*D* (mm)	36.8 (20.1)	30.3 (21.1)	0.091
MELD			0.703
MELD ≤ 15	29 (69.0%)	42 (76.4%)	
MELD = 15–30	9 (21.4%)	8 (14.5%)	
MELD > 30	4 (9.52%)	5 (9.09%)	
preTreat			0.319
No pre-treatment	23 (43.4%)	22 (32.8%)	
Pre-treatment	30 (56.6%)	45 (67.2%)	
*N*			0.109
*N* = 1	18 (48.6%)	41 (65.1%)	
*N* = 1–3	12 (32.4%)	18 (28.6%)	
*N* > 3	7 (18.9%)	4 (6.35%)	
Grade			0.559
1	8 (32.0%)	15 (44.1%)	
2	15 (60.0%)	18 (52.9%)	
3	2 (8.00%)	1 (2.94%)	
Asan			< 0.001
Exceeding	25 (47.2%)	10 (14.9%)	
Fulfilling	28 (52.8%)	57 (85.1%)	
AFP score			0.002
> 2	18 (40.9%)	8 (13.1%)	
≤ 2	26 (59.1%)	53 (86.9%)	
MC			0.001
Exceeding	30 (56.6%)	16 (23.9%)	
Fulfilling	23 (43.4%)	51 (76.1%)	
UTS			< 0.001
Exceeding	26 (49.1%)	8 (11.9%)	
Fulfilling	27 (50.9%)	59 (88.1%)	

Data are shown as the average and standard deviation (round brackets) for normally distributed data and as the median and quantiles (square brackets) for non-normally distributed data

*AFP* alpha-feto protein in ng/ml, *D* diameter of the largest tumor in mm, *MELD* model of end-stage liver disease, *N* number of tumors, *MC* Milan criteria, UTS up-to-seven criteria

## Discussion

HCC is the most studied primary tumor entity for the indication of liver transplantation. Because of the gap between the numbers of suitable donors and those of patients on the waiting list, transplantation should only be performed in patients who benefit most from organ transplantation. These patients can only be identified by clear guidelines and objective scoring systems.

The MC are widely used for the selection of patients and are also embedded in the MELD-based organ allocation programs in [1, 2]. Patients beyond the MC can be transplanted based on extended allocation policies [9, 10]. However, the limit for the exclusion of patients with advanced tumors from liver transplantation should be defined since the increase in tumor burden is related to a poor prognosis following transplantation [11, 12].

The current practice to prove the LTx indication is based on radiological findings such as tumor diameter and number of hepatic nodules. However, the accuracy of pre-transplant imaging in cirrhotic liver has been questioned in several studies when compared with posttransplant histopathology [8, 13].

To avoid these problems and to determine the real tumor load at the time of LTx, we analyzed our patients based on histopathological results. Then, we retrospectively classified the patients into established indication criteria for patients with HCC and assessed postoperative survival.

In the same manner, Lee et al. investigated the basic histopathological features of patients with HCC in living donor liver transplantation and introduced the so-called Asan criteria. Meeting the Asan criteria (largest tumor diameter ≤ 5 cm, number of tumors ≤ 6), they could achieve a 5-year overall survival of 76.3%, which was not significantly higher than the 5-year survival if meeting the MC in their population.

In our patients, who were transplanted following deceased donation, we observed similar survival curves for the patients fulfilling the MC or UTS or Asan criteria. Current data on the outcome of patients fulfilling the MC show overall 1-, 3-, and 5-year survival rates of 85–93%, 75–81%, and 68–73%, respectively [14, 15]. Our data were comparable to the international data showing 1-, 3-, and 5-year overall survival rates of 82%, 77%, and 69%, respectively, within the MC. Using extension criteria such as the AFP score, UTS, and Asan, we were able to show similar results [6, 8, 16, 17].

Interestingly, in our study, patients with an AFP score equal to or less than 2 showed a 5-year survival rate of 52%, a significantly worse long-term survival compared to patients in other groups. This difference is most likely due to the missing AFP data of 15 patients and consequently reduced statistical power.

An independent factor influencing postoperative survival after transplantation is the recurrence of HCC. In our analysis, HCC recurrence occurred after a median follow-up period of 28 months after transplantation. All of these patients died during the follow-up period. The univariate analysis determined a significant influence of the AFP level, number of tumors, maximum diameter of the largest tumor, and tumor grade on the outcome after LTx.

Whether AFP is a reliable marker is still an ongoing topic, and the cutoff AFP level as a contraindication for LTx has not been determined. In our study, only 21 patients with a maximum AFP level higher than 100 ng/ml and only ten patients with an AFP level higher than 500 ng/ml were transplanted, showing no unique survival or recurrence features (data not shown). Thus, we cannot assess a cutoff AFP level for contraindication for LTx in concordance with the current guidelines [5].

In our detection of HCC recurrence, established clinical scores, such as the MC, UTS criteria, Asan criteria, and AFP score, showed similar prediction for HCC recurrence following LTx. Concerning the two endpoint parameters together (overall survival and recurrence), the best performance with sensitivity and specificity was achieved when considering the UTS criteria.

Using UTS as the extension criteria for LTx, we could add 12 patients fulfilling UTS, which were beyond the MC and were theoretically excluded from regular allocation policy. Interestingly, the patients fulfilling the UTS but exceeding the MC in our population showed similar survival and recurrence rates compared to the patients fulfilling MC only. Thus, the extension of the MC using the UTS criteria for the indication for LTx must be considered in standard care.

The most important limitation of this study is its retrospective design. Secondly, the preoperative imaging techniques during the entire study period (1993 until 2012) were not exactly comparable. However, the sensitivity of preoperative imaging for focal lesions in cirrhotic tissue is still limited [18]. The consideration of tumor biology and the underlying disease needs to be included in the indication for LTx. New strategies, such as the detection of biomarkers in tumor tissue as well as in the sera of patients with HCC, could help identify new parameters for an indication for LTx in patients with HCC [19, 20].

## Conclusion

In conclusion, we were able to confirm the indication criteria for liver transplantation (MC, UTS criteria, Asan criteria, AFP score) based on the postoperative histopathological results in our population. In terms of specificity and sensitivity, the UTS criteria are superior to the other criteria in terms of overall and recurrence-free survival. Consequently, we consider using UTS criteria as the standard criterion for LTx indication.

## Data Availability

The datasets used and analyzed during the current study are available from the corresponding author on reasonable request.

## Abbreviations

MC: Milan criteria
LTx: Liver transplantation
HCC: Hepatocellular carcinoma
UTS: Up-to-seven
AFP: Alpha-fetoprotein
MELD: Model for end-stage liver disease
ALD: Alcoholic liver disease
